# Case for diagnosis. Cutaneous small vessel vasculitis (anti-proteinase 3 positive), fever, hemoptysis, and lung cavitation in an adult^☆^^☆☆^

**DOI:** 10.1016/j.abd.2020.06.009

**Published:** 2021-01-30

**Authors:** Luana Moraes Campos, Mariana Righetto de Ré, Priscila Neri Lacerda, Hélio Amante Miot

**Affiliations:** Department of Dermatology and Radiotherapy, Faculty of Medicine, Universidade Estadual Paulista, Botucatu, SP, Brazil

**Keywords:** Anti-neutrophil cytoplasmic antibodies, Anti-neutrophil cytoplasmic antibody-associated vasculitis, Tuberculosis, Vasculitis

## Abstract

Small vessel vasculitis with anti-proteinase antibodies 3 is an atypical clinical presentation of tuberculosis. The authors present the case of a 47-year-old male patient, with palpable purpura and palmoplantar hemorrhagic blisters, with subsequent dissemination. He presented severe pulmonary symptoms with cavitation, fever, hemoptysis, and high levels of anti-proteinase 3. Histopathological assessment of the skin revealed small vessel vasculitis; pulmonary histopathology showed granulomas with caseation. Bronchoalveolar lavage was positive for alcohol-acid-fast bacilli. In countries with a high prevalence of tuberculosis, the presence of autoantibodies in a patient with vasculitis, fever, and pulmonary cavitation requires investigation of infectious causes.

## Case report

**Figure 1:**
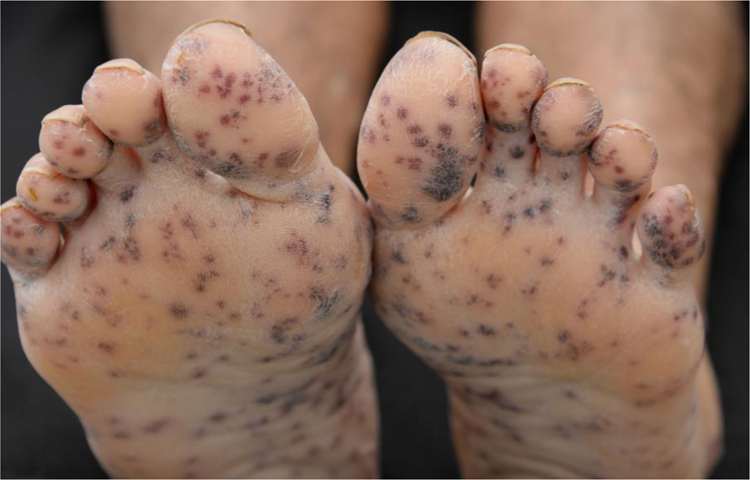
Multiple, bilateral palpable petechiae and purpura on the soles.

**Figure 2:**
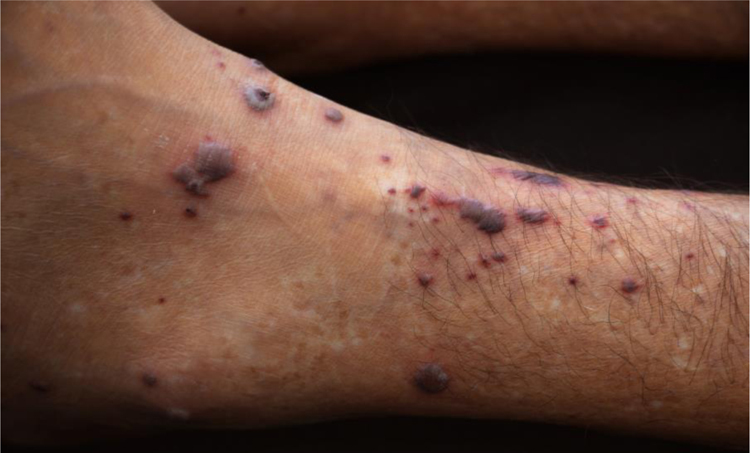
Palpable purpura and necrotic vesicles on the left lower limb.

**Figure 3:**
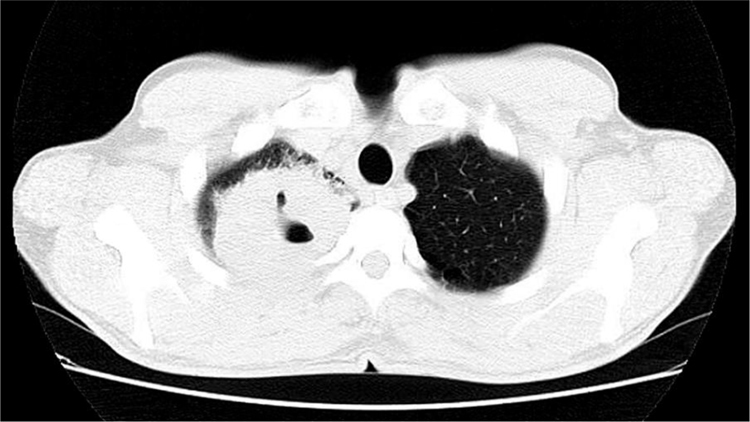
Computed tomography scan of the chest showing cavitation with a thick wall in the right upper lobe amid consolidations in other pulmonary areas.

47-year-old male, smoker, reported the appearance of petechiae, palpable purpura, and hemorrhagic blisters, on the soles and palms (Fig. 1) a week before, progressing to the lower and upper abdomen, and face (Fig. 2); he also reported bleeding in the eyes, nose, hemoptysis, and fever. Chest tomography revealed cavitation, with thick walls in the right upper lobe, and with sparse consolidations in other pulmonary areas (Fig. 3).

The laboratory tests demonstrated high levels of classic anti-neutrophil cytoplasmic antibody (c-ANCA: anti-proteinase 3 > 90 U/mL [reference values: < 5 U/mL]). Other viral serologies were negative, and the markers of inflammatory tests were very high: ESR 64 mm and CRP 19 mg/L.

**Figure 4:**
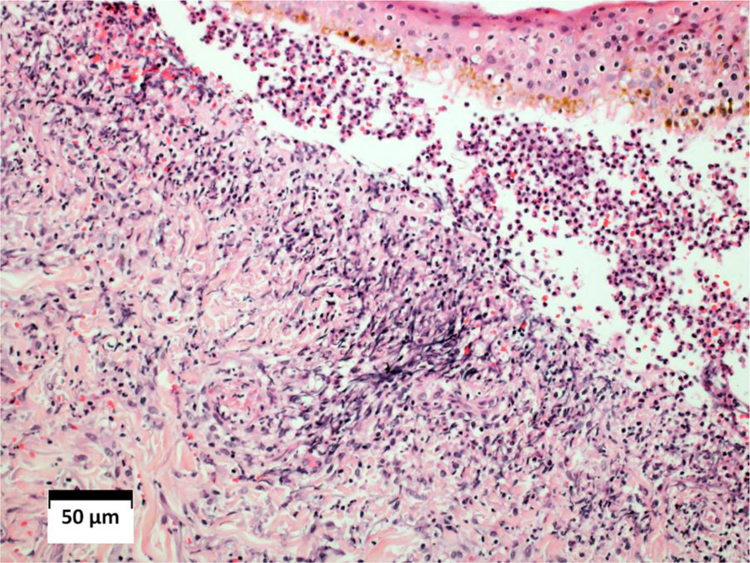
Histopathological examination of the skin showing intense diffuse inflammatory infiltrate in the dermis, red blood cells leakage, vascular walls infiltrated by neutrophils, and fibrinoid necrosis. Subepidermal vesicle, filled with red blood cells and neutrophils (Hematoxylin & eosin, ×10).

Histopathological examination of the skin revealed leukocytoclastic vasculitis, and direct immunofluorescence was negative with anti-IgM, anti-IgG, and anti-IgA antibodies. (Fig. 4). Pulmonary histopathology showed a granulomatous inflammatory process with central caseation. The bronchoalveolar lavage revealed three alcohol-acid fast bacilli (AAFB).

## What is your diagnosis?

a) Small vessel vasculitis secondary to tuberculosis
b) Granulomatosis with polyangiitis (Wegener's granulomatosis [WG])
c) Atypical mycobacteriosis with embolization
d) Association of tuberculosis with Wegener's granulomatosis

## Discussion

The most common manifestation of *Mycobacterium tuberculosis* infection is pulmonary; however, more than 10% of cases have an extrapulmonary presentation.^1^, ^2^ As the incidence of tuberculosis (TB) has been increasing worldwide, this affects the occurrence of atypical forms.

Skin lesions associated with TB are highly polymorphic, and can occur by direct action of bacilli, by inoculation, hematogenous propagation, deposition of immune complexes on the walls of small vessels (hypersensitivity vasculitis), or by the formation of antibodies against antigens in the host.^3^, ^4^, ^5^ Small vessel vasculitis secondary to TB is uncommon, with less than 20 cases reported in the literature. Three forms have been described: Henoch-Schönlein purpura, vasculitis secondary to rifampicin, and cutaneous leukocytoclastic vasculitis.^1^, ^3^, ^6^

TB can occur associated with other autoimmune diseases, such as WG, sharing similar clinical findings and histopathology.^2^, ^7^ Necrotic lung lesions in WG are radiologically similar to those observed in TB. It is worth mentioning that the literature presents two case reports in which these two diseases coexisted.^8^

Although ANCAs are considered to be markers of systemic vasculitis and are associated with WG and other autoimmune disorders, ANCA positivity has been demonstrated in infectious diseases such as TB, especially the c-ANCA pattern, increasing the possibility of diagnostic confusion with WG.^2^, ^7^, ^9^, ^10^

*M. tuberculosis* can stimulate the release of oxygen metabolites from activated neutrophils, which would release lysosomal enzymes in the early stages of infection, with the potential to induce autoantibodies against these components.^7^

In this case, the patient met diagnostic criteria for WG, and had high levels of c-ANCA, generating diagnostic doubt about the possibility of concomitant diseases. The absence of previous sinusopathy, asymmetry of pulmonary involvement, and lack of involvement of medium size vessels (livedo, ulcers, or necrosis of extremities), indicated an atypical form of TB.

The patient was submitted to an antituberculous regimen (RIPE) and corticosteroid therapy 1 mg/kg/day, with remission of the condition after one month of treatment, and normalization of c-ANCA after six months.

In countries with a high prevalence of TB, the presence of autoantibodies in a patient with vasculitis, fever, and pulmonary cavitation requires the investigation of infectious causes, especially tuberculosis, before admitting the diagnosis of WG.^2^, ^7^

## Financial support

None declared.

## Authors’ contribution

Luana Moraes Campos: Approval of the manuscript; drafting of the manuscript; effective participation in research orientation; effective participation in propaedeutics; literature review; critical review of the manuscript.

Mariana Righetto de Ré: Approval of the manuscript; drafting of the manuscript; effective participation in research orientation; effective participation in propaedeutics; literature review; critical review of the manuscript.

Priscila Neri Lacerda: Approval of the manuscript; drafting of the manuscript; effective participation in research orientation; effective participation in propaedeutics; literature review; critical review of the manuscript.

Hélio Amante Miot: Approval of the manuscript; drafting of the manuscript; effective participation in research orientation; effective participation in propaedeutics; literature review; critical review of the manuscript.

## Conflicts of Interest

None declared.
